# Mendelian randomization analyses of known and suspected risk factors and biomarkers for myasthenia gravis overall and by subtypes

**DOI:** 10.1186/s12883-024-03529-y

**Published:** 2024-01-18

**Authors:** Wenwen Wang, Wei Ge, Junling Feng, Manli Huang, Xihua Zhang, Jielai Xia, Ling Wang, Chen Li

**Affiliations:** 1 Department of Health Statistics, School of Preventive Medicine, Fourth Military Medical University, No.169 Changlexilu Road, Xi’an, Shaanxi Province 710042 P. R. China; 2 Department of Field and Disaster Nursing, Fourth Military Medical University, Xi’an, Shaanxi 710032 China; 3 Department of Neurological Intensive Care Rehabilitation, Xi’an International Medical Center Hospital, Xi’an, Shaanxi Province 710000 China

**Keywords:** Myasthenia gravis, Mendelian randomization, Cytokines, Intestinal microbiota, Serum metabolites

## Abstract

**Background:**

Myasthenia gravis (MG) is an autoimmune disease that affects neuromuscular junction. The literature suggests the involvement of circulating cytokines (CK), gut microbiota (GM), and serum metabolites (SM) with MG. However, this research is limited to observational trials, and comprehensive causal relationship studies have not been conducted. Based on published datasets, this investigation employed Mendelian Randomization (MR) to analyze the known and suspected risk factors and biomarkers causal association of MG and its subtypes.

**Methods:**

This research used two-sample MR and linkage disequilibrium score (LDSC) regression of multiple datasets to aggregate datasets acquired from the genome-wide association studies (GWAS) to assess the association of MG with 41-CK, 221-GM, and 486-SM. For sensitivity analysis and to validate the robustness of the acquired data, six methods were utilized, including MR-Egger regression, inverse variance weighting (IVW), weighted median, and MR-PRESSO.

**Results:**

The MR method identified 20 factors significantly associated with MG, including 2 CKs, 6 GMs, and 9 SMs. Further analysis of the factors related to the two MG subtypes, early-onset MG (EOMG) and late-onset MG (LOMG), showed that EOMG had a high overlap with MG in the intestinal flora, while LOMG had a greater similarity in CKs and SMs. Furthermore, LDSC regression analysis indicated that *Peptococcaceae*, oxidized biliverdin, and Kynurenine had significant genetic correlations with general MG, whereas EOMG was highly correlated with *Intestinibacter*, while LOMG had significant genetic associations with Kynurenine and Glucose.

**Conclusion:**

This research furnishes evidence for the potential causal associations of various risk factors with MG and indicates a heterogeneous relationship between CKs, GMs, and SMs with MG subtypes.

**Supplementary Information:**

The online version contains supplementary material available at 10.1186/s12883-024-03529-y.

## Introduction

Myasthenia Gravis (MG), a chronic autoimmune disease, is manifested with serum auto-antibodies against the nicotinic acetylcholine receptor (AChR) at the neuromuscular junction (NMJ) in most patients. The main clinical symptoms of MG are the changes in the NMJ and damage caused by antibodies, leading to partial or systemic abnormal muscle fatigue and weakness [1, 2]. Its incidence per annum is about 4–12 per million, whereas the global prevalence is 40‒180 per million people [3, 4]. Depending on whether the patient is over 50 years old at the time of onset, MG can be divided into late-onset MG (LOMG) and early-onset MG (EOMG) subtypes [5]. With the rising prevalence and morbidity in the elderly population, especially LOMG, the mortality rate of MG has markedly increased [6]. Adjuvant antibody testing is time-consuming, relatively expensive, not readily available, and has a high false-negative result rate [1]. Furthermore, although immunosuppressive and conventional hormonal therapies can relieve symptoms, the MG recurrence rate is still quite high, and no consensus has been reached on the ideal therapeutic algorithm for MG [7]. Therefore, early identification and development of more specific therapies are critical for MG patient’s better quality of life.

A thorough understanding of the pathogenesis of MG is helpful for the diagnosis and treatment. Some studies have found genes related to genetic susceptibility to MG, such as HLA-DQ5, CTLA-4, etc., but the etiology of MG immune-related needs further study. The epidemiological literature has indicated the associations of MG with cytokines (CK) [8, 9], gut microbiotas (MG) [10, 11], serum metabolites (SM) [12, 13], circulating inflammatory proteins (CIF), and immune cell signatures (ICS). The causal associations of many of these remain undetermined, as most evidence was acquired from observational studies, which are often limited by recall, selection, confounding, reverse causation biases, and measurement errors.

In epidemiologic research, Mendelian randomization (MR) assessment has widely been used for assessing causal inference [14]. The MR analysis employs genetic variants as instrumental variables (IVs) to calculate exposures of interest for association analysis of disease outcomes. Since during gametogenesis, genetic alleles are classified randomly, MR resembles randomized clinical trials, minimizing or eliminating the potential for confusion and reverse causality bias common in traditional epidemiological research [15]. The associations of genetic variants with MG have been assessed in various genome-wide association studies (GWAS). The molecular analyses of peripheral T-cell traits have determined three T-cell traits as causally protective factors against MG [16]. Currently, studies for the evaluation of a large number of known and suspected MG risk factors are lacking. MR analysis could serve as an efficient tool for exploring causal relationships between new biomarkers and MG risk using the existing large data sets, which is rarely done in the field. Using the same methodology to assess these risk factors in one study will help compare the association strength and furnish a comprehensive understanding of MG etiology. Understanding the relationship between MG’s intrinsic subtypes and these risk factors may provide additional insights into its etiology and biology.

Therefore, this large MR research comprehensively elucidated the associations of 41-CK, 221-GM, and 486-SM with MG risk and assessed their associations with the EOMG and LOMG subtypes.

## Materials and methods

### Study design

**Fig. 1:**
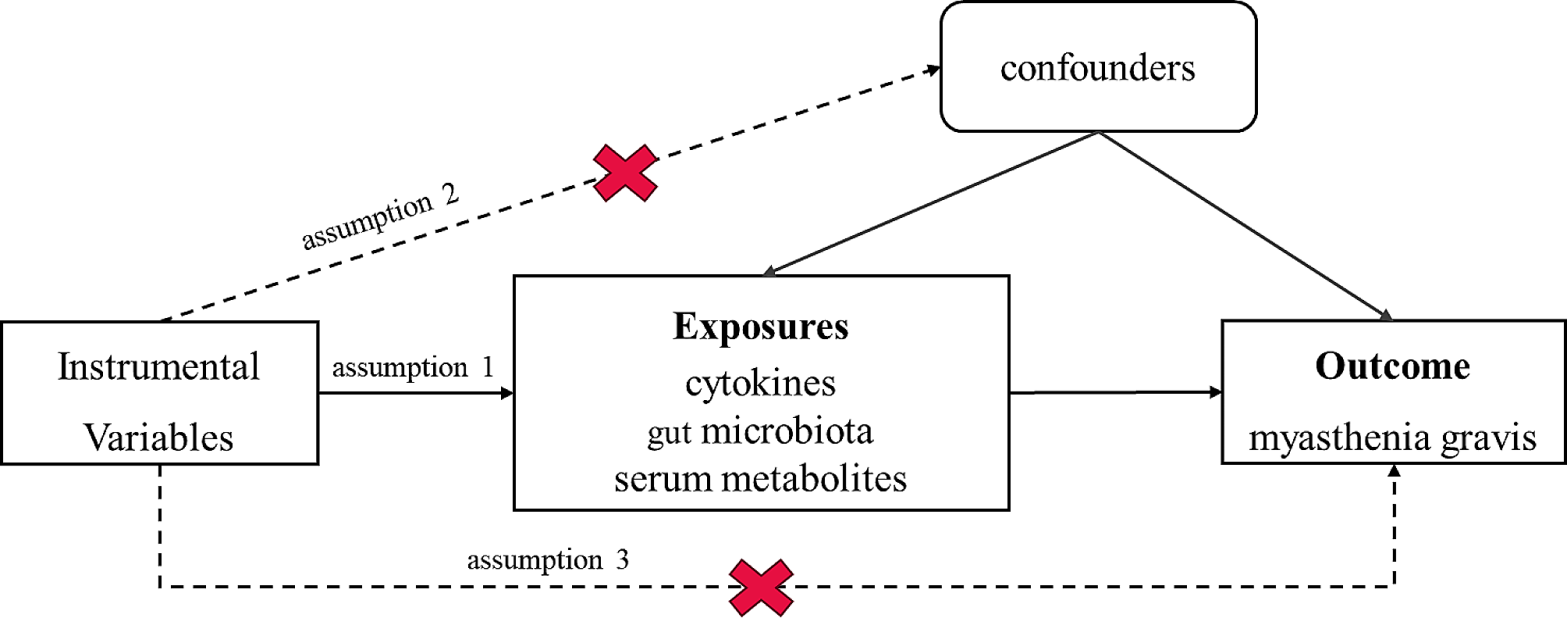
The primary design and assumptions of the bidirectional Mendelian randomization research: (1) instrumental variables are closely linked with exposure, (2) instrumental variables and any confounding factors were independent, and (3) instrumental variables could influence outcomes only *via* exposure. SNP: single-nucleotide polymorphism

Figure 1 indicates the assumptions of this MR study. This research followed three basic assumptions: (1) genetic variants were related to exposure, (2) confounders and genetic variants are not associated, and (3) outcomes were only linked with genetic variants through exposure. The inverse variance-weighted (IVW) method was applied as the primary method, which equals a weighted regression of the SNP outcome effect on SNP exposure with a fixed y-intercept of zero [17]. The simple mode, weighted median, and MR-Egger test were determined to enhance the robustness of the data. Furthermore, to assess the robustness or potential bias of results, sensitivity analyses were carried out. These included the MR-Egger intercept test for pleiotropy, Cochrane’s Q test for heterogeneity, and leave-one-out analyses [18]. The heterogeneity analysis determined the differences between each IV, whereas the pleiotropy assessed the presence of horizontal pleiotropy.

### Data sources

The data were sourced from GWAS. Table 1 depicts the detailed information of various GWAS datasets. This investigation employed a two-sample MR method to elucidate the causal relationship of MG with 41 CK, 221 gut microbiotas (GM) (genus level), and 486 SM. In detail, the CK dataset comprised the meta-analysis summary statistics of GWAS on inflammatory CKs performed on 3 Finnish cohorts (FINRISK 1997, YFS, and 2002) [19]. For gut microbial taxa, the summary statistics were acquired from a multi-ethnic large-scale GWAS meta-analysis carried out by the MiBioGen consortium and included 19,790 participants from 18 cohorts [20]. MiBioGen is the most comprehensive effort for elucidating host-genetics vs. microbiome correlation on a population scale and comprises 211 taxa (131 genera, 35 families, 20 orders, 16 classes, and 9 phyla). The 486 human SM genetic datasets were acquired from the Metabolomics GWAS Server (http://metabolomics.helmholtz-muenchen.de/gwas/), which included 7824 European participants (KORA and TwinsUK cohorts) [21]. The MG-related genetic variants were identified from 3 datasets. The primary analysis MG dataset was obtained from the current largest meta-GWAS performed in Italy and the US (1,873 patients vs. 36,370 controls) [22]. Only MG patients who were anti-AChR antibody-positive (AChR^+^) were selected for this study, and those who were positive for muscle-specific kinase (MuSK^+^) antibodies were excluded. To specify the age-dependent genetic heterogeneity, summary statistics of LOMG (1,278 patients vs. 33,652 controls) and EOMG (595 patients vs. 2,718 controls, aged ≤ 40 years) were separately analyzed. The validation datasets included UK (http://www.nealelab.is/uk-biobank) (234 patients vs. 324,074 controls) and FIN (https://gwas.mrcieu.ac.uk/datasets/finn-b-G6 MYASTHENIA/) (232 patients vs. 217,056 controls) Biobanks [23]. The MG phenotype was identified using questionnaires completed by the participants; data on MG subtypes were not applicable. All original articles had received ethical approval and the participants had provided informed consent. This investigation included individuals of European ancestry to minimize population stratification bias.

**Table 1:** Summary of the source GWAS datasets used in this study

Datasets	Phenotype	Data sources	Cases	Ancestry
Exposure 1	41 kinds of cytokines	University of Bristol	8,293	European
Exposure 2	221 gut microbiotas	MiBioGen	19,790	European
Exposure 3	486 serum metabolites	Metabolomics	7,824	European
Outcome 1	General myasthenia gravis	IMGGC	1,873/36,370	European
Outcome 2	Early-onset myasthenia gravis	IMGGC	595/3,313	European
Outcome 3	Late-onset myasthenia gravis	IMGGC	1,278/33,652	European

### SNP selection criteria

To identify the most representative, unbiased, and key genetic variables, various quality control procedures were carried out to assess eligible crucial single nucleotide polymorphisms (SNPs). (1) Based on minor allele frequency (MAF) > 0.01 and genome-wide significance (*P* < 5 × 10^8^), SNPs related to the exposures were identified. (2) Considering that multiple SNPs might be present adjacent in linkage disequilibrium status, the Clumping step was carried out with ${r}^{2}$ < 0.001, and the window size = 10,000 kb based on the European 1000 Genome Project, and those with the lowest *p*-value were retained [24]. (3) The exposure SNPs were isolated from the outcome GWAS summary data. In case a specific exposure SNP was missing in the outcome GWAS, a proxy SNP in linkage disequilibrium was employed with the exposure SNP (minimum LD ${r}^{2}$ = 0.8). (4) Ambiguous SNPs, from which effect allele could not be assessed, were eliminated by harmonizing the outcome SNPs and exposure. Palindromic SNPs in the original datasets were reviewed separately to avoid undesired reverse effects. F-statistics evaluated the strength of the genetic instrument and a genetic variant with < 10 *F-statistics* for a specific IV was termed weak and not included in the MR analysis. The *F*-statistic was measured as follows:

$$F={R}^{2}\times (N-k-1)/k(1-{R}^{2})$$

where $N$ = the sample size, $k$ = the number of contained SNPs, and ${R}^{2}$ = the proportion of exposure variance explained by a particular genetic variant. *R*^*2*^ was assessed as follows:

$${R}^{2}= 2\times {\beta }^{2}\times \text{E}\text{A}\text{F}\times (1-\text{E}\text{A}\text{F})$$

where $\beta$ = the estimated effect of the genetic variant, and EAF = the effect allele frequency [25]. Table 1 and S1 provide details of the genetic instruments used.

### Two-sample mendelian randomization analysis

**Fig. 2:**
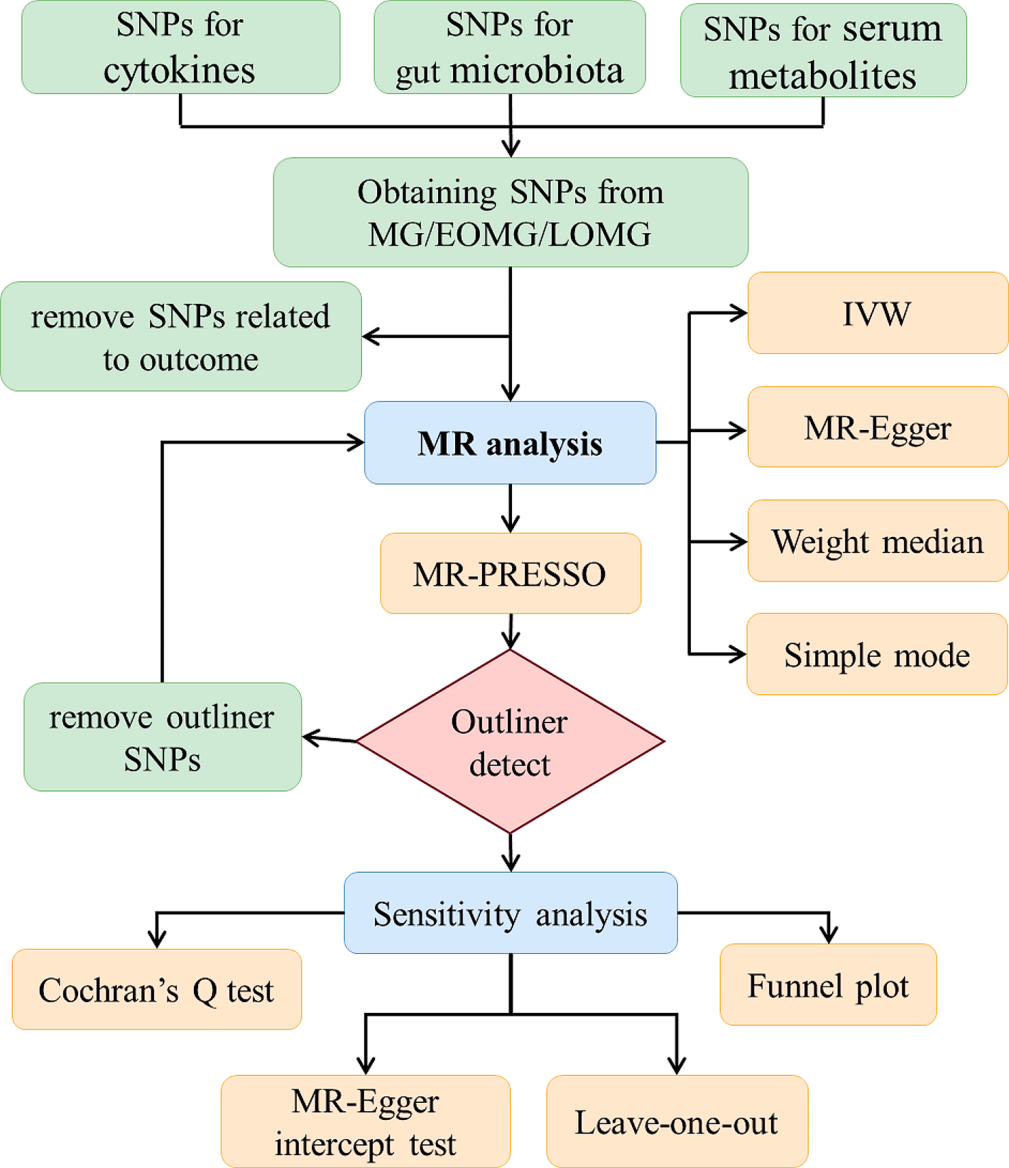
The Mendelian randomized study workflow revealed causal relationships between cytokines, gut microbiotas, and serum metabolites and the risk of myasthenia gravis. MR-PRESSO: MR pleiotropy residual sum and outliers, MR: Mendelian randomization, IVW: inverse variance weighting

Figure 2 depicts the workflow of a two-sample MR analysis. Various MR methods were employed to elucidate the causal impact of identified exposure variables on the outcome. The primary analysis method was Inverse variance weighting (IVW), and all causality estimates were converted into odds ratios (OR) of dichotomous phenotypic outcomes. MR Egger, weight median [26], simple model, and MR-PRESSO [27] were performed at the same time. Sensitivity analysis was performed, which included the MR-Egger intercept test for pleiotropy [18], Cochrane’s Q test for heterogeneity, and leave-one-out analyses to assess the robustness and potential bias in the results. Heterogeneity tests were carried out to examine the differences between each IV, and pleiotropy tests examined the presence of horizontal pleiotropy. Finally, each exposure’s statistical power was elucidated using a two-sided type-I error rate α = 0.05 [28].

### Evaluation of generation of genetic correlation and directionality

Linkage Disequilibrium Score (LDSC) regression was implemented to elucidate the genetic association across exposures to explore the probability of a shared genetic architecture [29]. Pre-calculated LD scores of European participants in the 1000 Genomes project were used for high-quality HapMap3 SNPs. Through LDSC regression, the genetic correlation (rg) between the two traits was evaluated by combining GWAS summary statistics and the LD scores with a regression model. Furthermore, the Steiger test was performed to avoid the resulting bias caused by reverse causality [30].

### Metabolic pathway analysis

For metabolic pathways analysis, web-based Metaconflict 5.0 (https://www.metaboanalyst.ca/) [31] was used. With the help of functional enrichment and pathway analysis modules, potential groups of metabolites or pathways linked with outcome biological processes were identified. This investigation used the Kyoto Encyclopedia of Genes and Genomes (KEGG) database, and for pathway analysis, the significance level was set at 0.10.

### Statistical analysis

Two Sample MR package [17] was used for MR analysis in R version 4.2.2. Other packages for data processing and graph generation included ieugwasr, tidyverse [32], readr, ldscr, and forestplot.

## Results

The detailed MR analysis results on the association of 41 CKs, 221 GMs, and 486 SMs exposures with overall MG, EOMG, and LOMG are presented in Supplement File 1–3. Furthermore, the MR analysis results of replicate data of exposure factors and overall MG are displayed in Supplement File 4–5.

### Associations with general myasthenia gravis risk

**Fig. 3:**
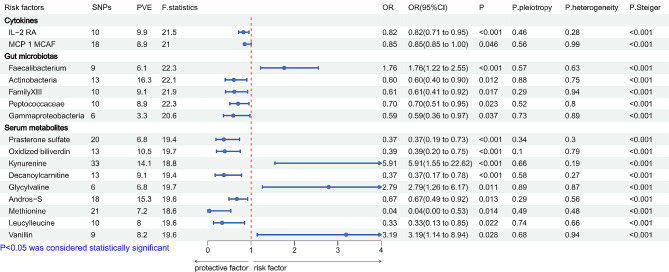
Forest plot for the causal effect of cytokines, gut microbiotas, and serum metabolites on the risk of MG derived from inverse variance weighted (IVW); MG: myasthenia gravis; OR, odds ratio; CI, confidence interval

Figure 3 shows the CKs, GMs, and SMs significantly associated with general MG risk. MR analysis revealed that the quantity of SNPs for all exposures ranged from 6 to 33, the proportion of variance explained (PVE, %) ranging from 3.3 to 25.9, and *F*-statistics for all exposures were > 10. IVW analysis showed that among the CK exposure factors, IL-2 RA and MCP 1 MCAF were substantially related to a reduced risk of general MG. In the GMs, six types of bacteria were significantly correlated with general MG. An increased level in *Faecalibacterium* and *MollicutesRF9* were markedly linked with an increased risk of general MG, while *Actinobacteria*, *FamilyXIII*, *Peptococcaceae*, and *Gammaproteobacteria* were associated with a notably decreased risk of general MG. A total of nine SMs were significantly associated with general MG. Three of these factors (kynurenine, glycylvaline, and vanillin) were linked with an increased general MG risk, whereas six factors (prasterone sulfate, oxidized bilirubin, decanoylcarnitine, androsterone sulfate, methionine, and leucylleucine) were substantially associated with reduced risk of general MG (Fig. 1). Furthermore, it was found that metabolites significantly related to general MG were mainly enriched in methionine and cysteine metabolism (*p* = 0.08) and chlorophyll and porphyrin metabolism (*p* = 0.07) (Supplementary File 1). The estimated value of associations obtained from MR-PRESSO and MR-Egger regression analysis were consistent with the data of the IVW method (Supplementary Table 1). The MR-Egger intercept test, Cochrane’s Q test, and LOO analysis revealed no strong evidence of heterogeneity and directional horizontal pleiotropy (Fig. 1, Supplementary Fig. 1). Steiger’s directionality analysis revealed that all the data were consistent with the correct result orientation exposure.

### Associations with early-onset and late-onset MG risk

**Fig. 4:**
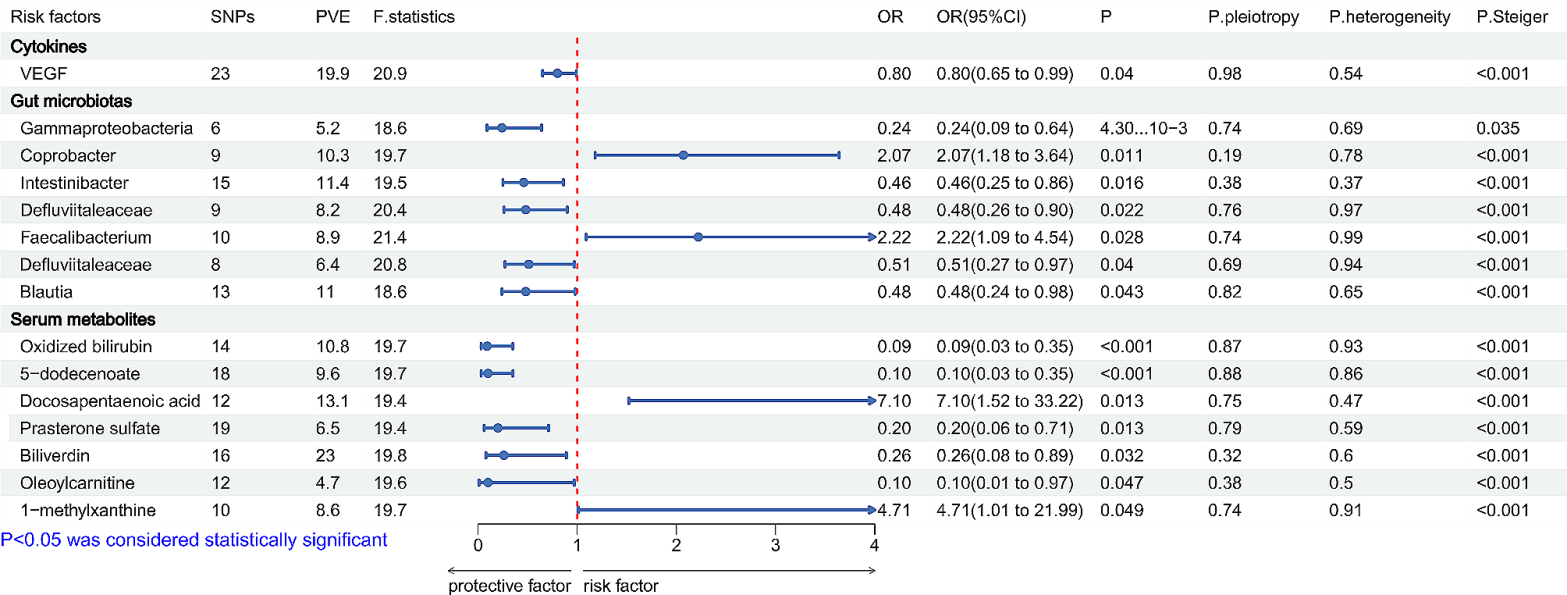
Forest plot for the causal effect of cytokines, gut microbiotas, and serum metabolites on the risk of EOMG derived from inverse variance weighted (IVW); EOMG: early-onset myasthenia gravis; OR, odds ratio; CI, confidence interval

**Fig. 5:**
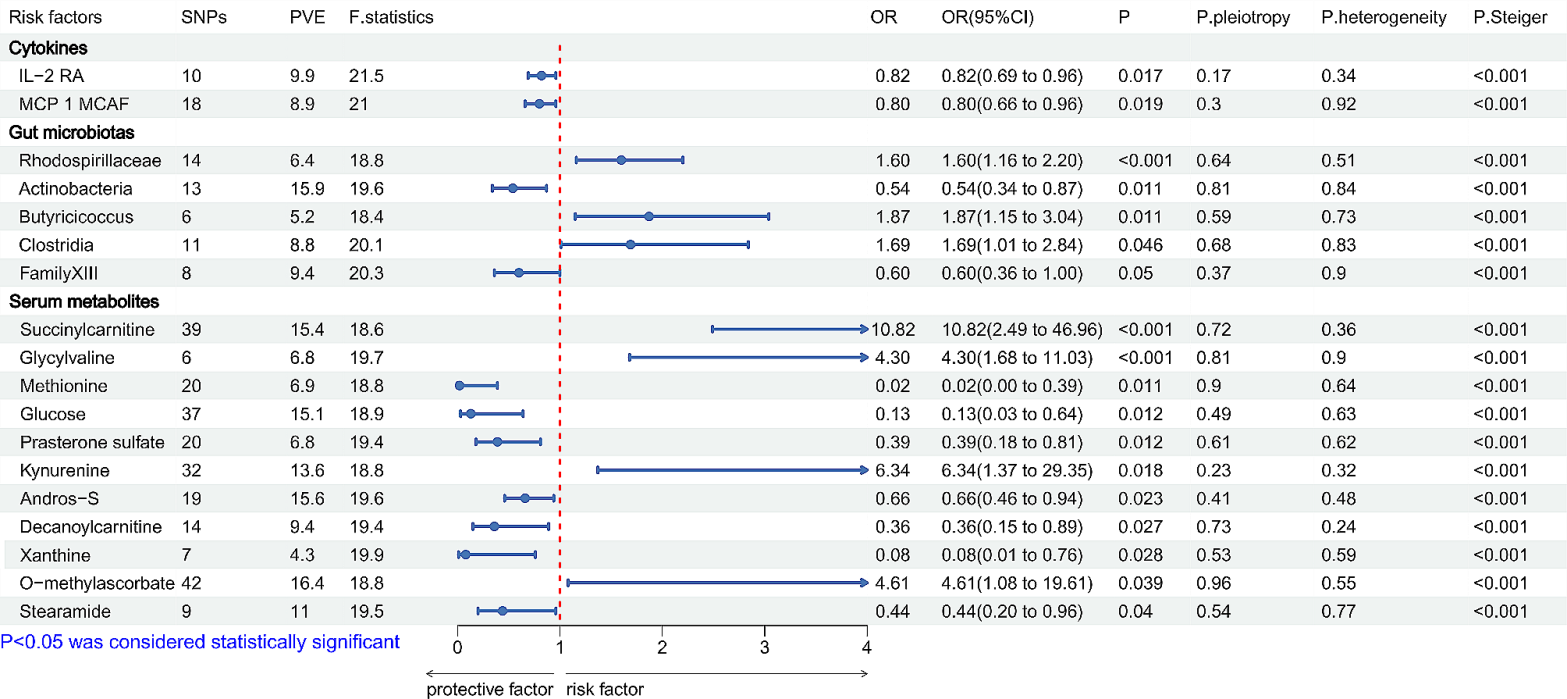
Forest plot for the causal effect of cytokines, gut microbiotas, and serum metabolites on the risk of EOMG derived from inverse variance weighted (IVW); LOMG: late-onset myasthenia gravis; OR, odds ratio; CI, confidence interval

As Figs. 4 and 5 showed, fifteen and eighteen significant exposure factors were identified associated with EOMG and LOMG respectively, with SNP numbers ranging from 6 to 42 and PVE (%) values ranging from 3.5 to 23.0. In the analysis of CKs, EOMG was significantly correlated with VEGF, while LOMG was significantly correlated with IL-2 RA and MCP 1 MCAF, indicating protective effects. Furthermore, the results of LOMG were more consistent with general MG while compare with EOMG. The GM analysis revealed that EOMG and LOMG were markedly correlated with seven and five species, respectively. In EOMG, the OR value calculation results showed that *Gammaproteobacteria*, *Intestinibacter*, *Defluviitaleaceae*, *Defluviitaleaceae*, and *Blautia* were protective factors, while *Coprobacter* and *Faecalibacterium* were risk factors. Meanwhile, in LOMG analysis, *Actinobacteria* and *FamilyXIII* showed protective effects, whereas *Rhodospirillaceae*, *Butyricicoccus*, and *Clostridia* were pathogenic factors. Moreover, *Gammaproteobacteria* was indicated to serve as a protective factor in EOMG and general MG, while *Faecalibacterium* was a significant pathogenic factor. For LOMG and general MG, *Actinobacteria* and *FamilyXIII* were identified as protective factors. In the exploration of SM, seven and eleven metabolites were found in EOMG and LOMG, respectively, which were significantly correlated with outcome. Additionally, it was found that choline, docosapentaenoic acid, and 1-methylxanthine were risk factors when EOMG was the outcome event, and oxidized bilirubin, 5-dodecenoate, prasterone sulfate, biliverdin, and oleoylcarnitine were protective factors. The LOMG analysis indicated four metabolites as risk factors, including succinylcarnitine, glycylvaline, kynurenine, and O-methylascorbate, and seven were protective factors, including methionine, glucose, androsterone sulfate, decanoylcarnitine, xanthine, prasterone sulfate,and stearamide. Compared with the results of serum metabolite analysis of general MG showed that oxidized bilirubin, prasterone sulfate, and oleoylcarnitine were common significant causal factors for EOMG, whereas common significant associations factors for LOMG included glycylvaline, methionine, prasterone sulfate, kynurenine, androsterone sulfate, and decanoylcarnitine. Notably, prasterone sulfate was significantly associated with all three outcomes. MR-PRESSO results were consistent with the IVW method (Supplementary Tables 2–3). The test of pleiotropy and heterogeneity did not show statistical significance. Metabolic pathway analysis found that metabolites significantly associated with EOMG were enriched in the porphyrin and chlorophyll metabolism (*p* = 0.003) and caffeine metabolism (*p* = 0.03) pathways, and those associated with LOMG were enriched in the glycolysis /gluconeogenesis (*p* = 0.08) pathway (Supplementary files 2 and 3). LOO analysis demonstrated that the MR results were robust and reliable (Supplementary Figs. 2 and 3).

Overall, the results of MR, general MG, and EOMG analyses provided more significant causal factor coverage in the GM, and LOMG and general MG had more significant associations in CKs and SM. This suggests that different factors may be highly correlated with MG at different onset stages.

### Results of genetic correlation analysis

The LDSC regression revealed that *Peptococcaceae* (rg = 0.653, se = 0.260, *p* = 0.012), oxidized biliverdin (rg = 0.296, se = 0.142, *p* = 0.037), and kynurenine (rg = 0.228, se = 0.105, *p* = 0.030) have a significant positive correlation with general MG. Whereas EOMG was positively correlated with *Intestinibacter* (rg = 1.240, se = 0.402, *p* = 0.002) and kynurenine (rg = 0.245, se = 0.111, *p* = 0.016) and glucose (rg = 0.341, se = 0.144, *p* = 0.017) demonstrated a positive genetic correlation with LOMG (Supplementary File 1–3).

## Discussion

Myasthenia gravis belongs to the largest group of neuromuscular junction disorders. It is a B cell-mediated autoimmune disease caused by pathogenic autoantibodies against the postsynaptic muscle endplate and manifests with muscle fatigue and weakness [5, 33]. Although the prognosis for MG patients is good, and the treatments include immunosuppressive, symptomatic, and supportive treatment, 10‒15% of patients show forms of uncontrollable disease and almost all patients require long-term medication [34]. MG patients are categorized into subgroups based on prognosis, optimum therapy, and diagnosis. MG is a reversible disorder that can be treated with intensity and optimism. Therefore, identifying high-risk factors according to subgroups will help further understand their pathogenesis and the development of better prevention and treatment.

It has been suggested that the incidence of MG is increasing because of the alterations in external causative factors, including infection and diet [35]. Previous reports have found that MG may be associated with CKs, GMs, and SMs. Furthermore, we have summarized the relevant methods and conclusions of the MR studies related to MG in the past five years (Table 2). This research is the first to elucidate the genetic correlations and causalities between CKs, GMs, and SMs and the risk of general MG, EOMG, and LOMG using the GWAS dataset. First, the genetic instrumental variables, which were substantially associated with different outcomes, were selected through MR and sensitivity analyses. Furthermore, an enrichment analysis of metabolite pathways that were significantly associated was conducted, and LDSC was employed to analyze the genetic correlation between these significantly associated exposure factors and outcomes.

**Table 2:** Summary of research methods and conclusions related to MG

Exposures	Methods	Conclusions
Vitamin D	Bidirectional two-sample MR	Circulating vitamin D levels had no causal effect on MG and MG had no causal effect on circulating vitamin D [36].
COVID-19	LDSC; Two-sample MR	No evidence of a genetic correlation or causal relationship among COVID-19 susceptibility, hospitalization, severity, and MG [37].
Ischemic stroke (IS)	Bidirectional two-sample MR	Bidirectional MR analysis did not provide evidence to support a causal relationship between genetically predicted MG and IS [38].
PCSK9 inhibitor	Two-sample MR	No evidence of a genetic correlation or causal relationship among PCSK9 inhibitor and MG [39].
Physical activity and sedentary behavior	LDSC; two-sample MR; MVMR	Findings support a causal effect of sedentary behavior as measured by LST on MG [40].
Five autoimmune diseases	Bidirectional two-sample MR	Results supported a bidirectional causal association between MG and SLE/T1DM [41].
Gut microbiota	Bidirectional two-sample MR	Research results yielded evidence of a causality connection in both directions between gut microbiota and myasthenia gravis [42].
T-cell traits	Two-sample MR	Three T-cell traits were identified to be causally protective for MG risk: (1) CD8 on terminally differentiated CD8^+^ T cells; (2) CD4^+^ regulatory T proportion in T cells; (3) HVEM expression on total T cells and other eight T-cell subtypes (e.g., naïve CD4^+^ T cells) [16].

LDSC: linkage disequilibrium score; MVMR: multivariable extension analyses; LST: leisure screen time; SLE: systemic lupus erythematosus; T1DM: type 1 diabetes

The results identified 20 significant exposure factors in general MG, including two CKs, six intestinal flora, and nine SMs. In the analysis of MG with different onset times, slightly different results were found from general MG, with fifteen and eighteen remarkably associated exposure factors found in EOMG and LOMG, respectively. Further analysis demonstrated that EOMG and general MG have a significant similarity in IM-related factors, while LOMG has a high degree of overlap in CKs and SMs, which indicates that there are differences in exposure factors related to the high risk of MG in different onset periods.

### Myasthenia gravis and cytokines

The literature suggests that in MG, various CKs and lymphocytes induce pathogenic inflammation and autoantibodies at the neuromuscular junction. Therefore, treatment targeting CKs could benefit patients with refractory MG [43–47]. . Furthermore, it has been indicated that serum IL-21, a follicular Th cell-related CK [41], is linked with elevated QMG score in MG patients, which decreases after steroid treatment [48]. Moreover, the rate of IL-10 is increased after the treatment of MG [49]. IL-6, Th 22, and TNF-α have been reported to take part in the pathogenesis and treatment of MG [50, 51]. This research significantly associated IL-2 RA and MCP 1 MCAF with general MG and LOMG risk. However, VEGF was more significantly associated with EOMG. It has been found in many studies that IL-2 is elevated in the serum of MG patients, and the receptor of IL-2 has great potential in the current field of immunotherapy research [52, 53], which validates the results of this investigation. However, MCP1, MCAF, and VEGF have not been reported in MG studies, which may be the direction for further experimental verification.

### Myasthenia gravis and intestinal microbiota

Recently, it has been suggested that alterations in the gut microbiome are associated with the incidence and development of MG, and some changes in the abundance of gut microbes may weaken or promote an immune response, suggesting potential diagnostic biomarkers for MG prevention, diagnosis, and treatment [54, 55]. *Qiu et al.* indicated that in comparison with the healthy cohort, the gut microbiota abundance of bacterial taxa was altered in the MG group, with a sharp decrease in microbial abundance, especially in *Clostridium* [56]. In addition, in MG patients, microbial dysbiosis was associated with serum levels of inflammatory biomarkers. *Moris et al.* found that the relative proportions of *Verrucomicrobiaceae* and *Bifidobacteriaceae* were lower in MG patients, while *Bacteroidetes* and *Desulfovibrionaceae* were increased [57]. However, the causal relationships between intestinal flora and MG have not been studied. Here, it was found that EOMG and general MG have more similar GM biomarkers, indicating that intestinal flora might be crucially involved in the early development of MG, which is conducive to the early diagnosis of MG. Many studies have indicated that *Faecalibacterium*, *Actinobacteria*, and *Clostridia* are related to the occurrence and development of MG, which is verified in this research [57–59].

### Myasthenia gravis and serum metabolites

In recent years, metabolomic analysis has significantly increased the understanding of MG [60]. *Derrick Blackmore et al.* identified 5 library-matched unique and 7 putative metabolites, including 2-methylbutyrylglycine, 3-hydroxybenzoic acid, and 3-methoxytyramine [61]. *Yonghai Lu* used LC-MS combined with multivariate statistical analysis to identify and classify SM associated with MG. Different metabolic profiles were observed at EOMG and LOMG, and 9 biomarkers, including gamma-aminobutyric acid and 1-phosphosphingosine, were identified [62]. This MR analysis identified 486 SM; of these, 10 to 12 metabolites were substantially associated with general MG, EOMG, and LOMG, respectively, in addition to the as-yet-undefined metabolites. Furthermore, kynurenine showed a significant genetic correlation with general MG and LOMG in LDSC analysis, consistent with previous studies [63].

### Limitations

This investigation has several limitations. (1) Although subgroup analyses of EOMG and LOMG were performed separately, other MG subgroups, including thymoma- and MUSK-associated MG, antibody-negative generalized MG, LRP4-linked with MG subtypes, and ocular MG, were not analyzed. However, the incidence of these subtypes is very low (2–4%), and they have no available GWAS data. Further analysis of subgroup risk factors can be conducted in the future. (2) Although the selected SNPs indicated a strong association, shared SNPs cannot be considered exact proxies for exposure as they do not adequately clarify the overall variance in complicated traits. (3) Research on other ethnicities should be performed to verify the study conclusions since all the GWAS data was on patients of European ancestry.

## Conclusion

In summary, this comprehensive two-sample MR study employed recent instrument variables to support potential CKs, GMs, and SMs markers associated with MG risk, stratified by MG subtypes. Furthermore, this work furnishes evidence on various risk factors associated with MG risk, varying with the timing of MG onset, highlighting the heterogeneity of the disease, which may help identify biomarkers for MG screening and improve understanding of MG occurrence.

### Electronic supplementary material

Below is the link to the electronic supplementary material.

Supplementary Material 1

Supplementary Material 2

Supplementary Material 3

Supplementary Material 4

Supplementary Material 5

Supplementary Material 6

## Data Availability

The original contributions presented in the study are included in the article or Supplementary Material. Further inquiries can be directed to the corresponding authors.
