# Supplementary material for: Mendelian randomization analyses of known and suspected risk factors and biomarkers for myasthenia gravis overall and by subtypes
Source: BMC Neurol. 2024 Jan 18;24:33. doi: 10.1186/s12883-024-03529-y (PMC10795466; doi:10.1186/s12883-024-03529-y)
Supplement: Supplementary file 6 — Supplementary Material 6 [file 12883_2024_3529_MOESM6_ESM.docx]

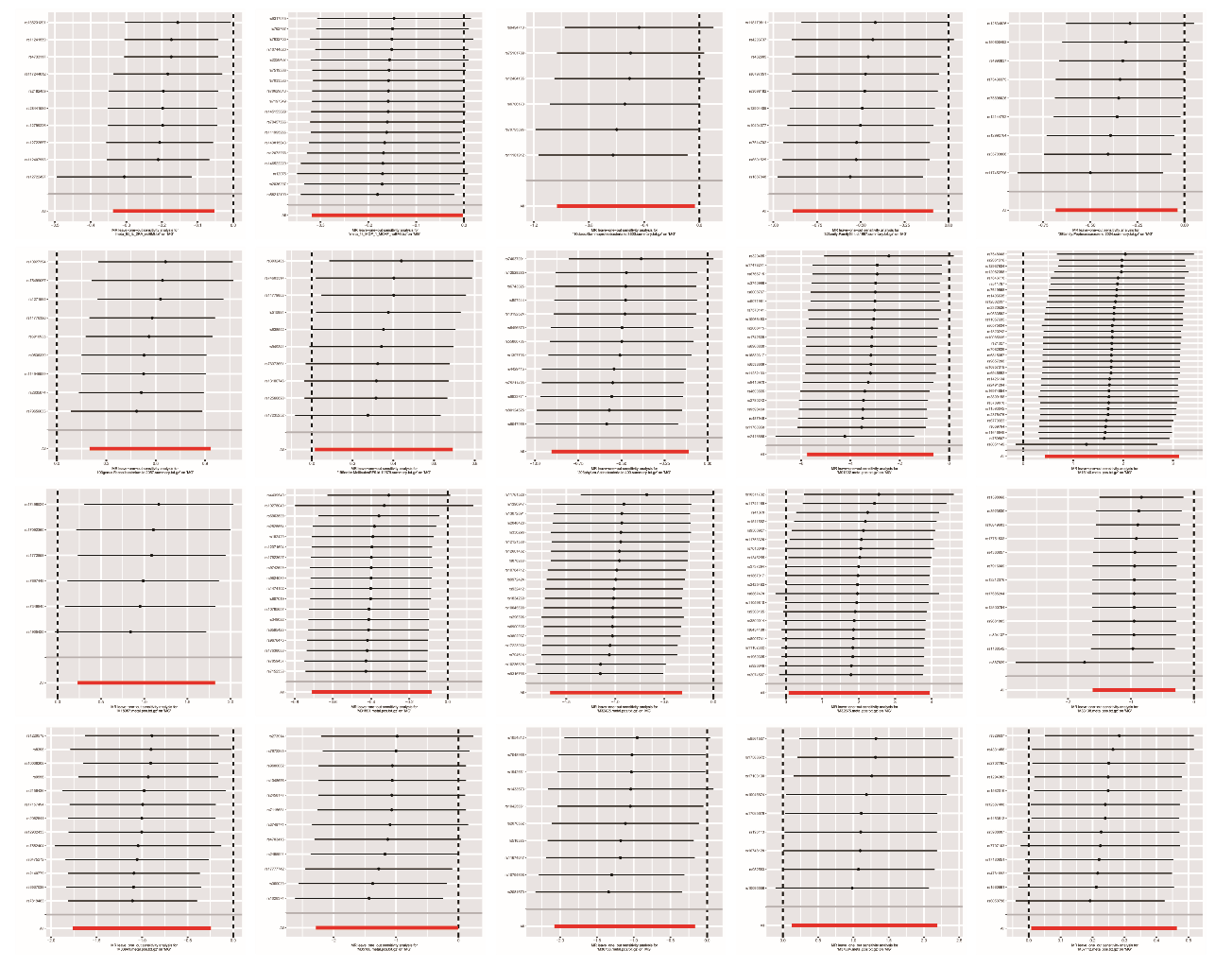


Supplementary Figure 1 Summary figures of LOO analysis results of significantly correlated exposure factors and general MG.


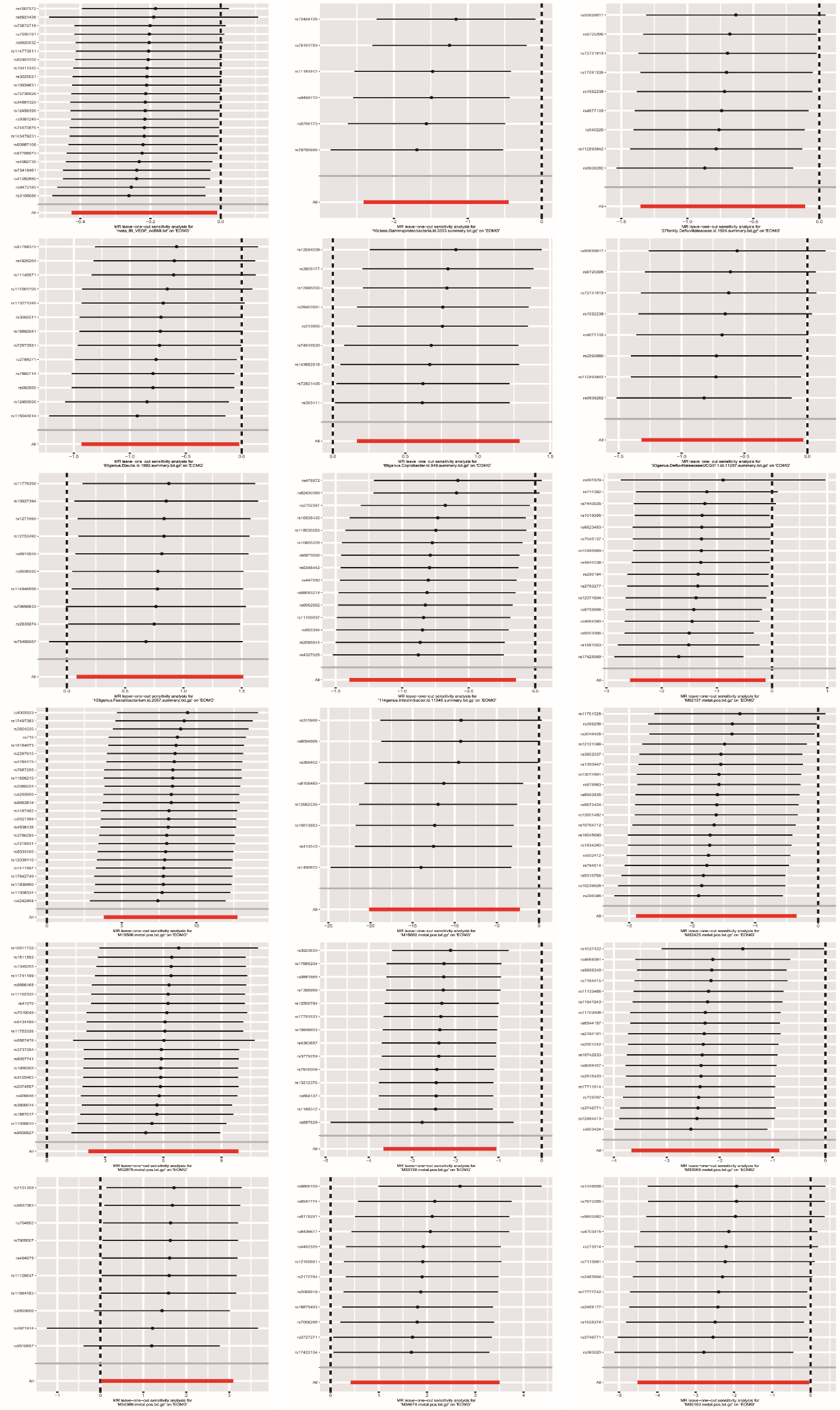


Supplementary Figure 2 Summary figures of LOO analysis results of significantly correlated exposure factors and EOMG.


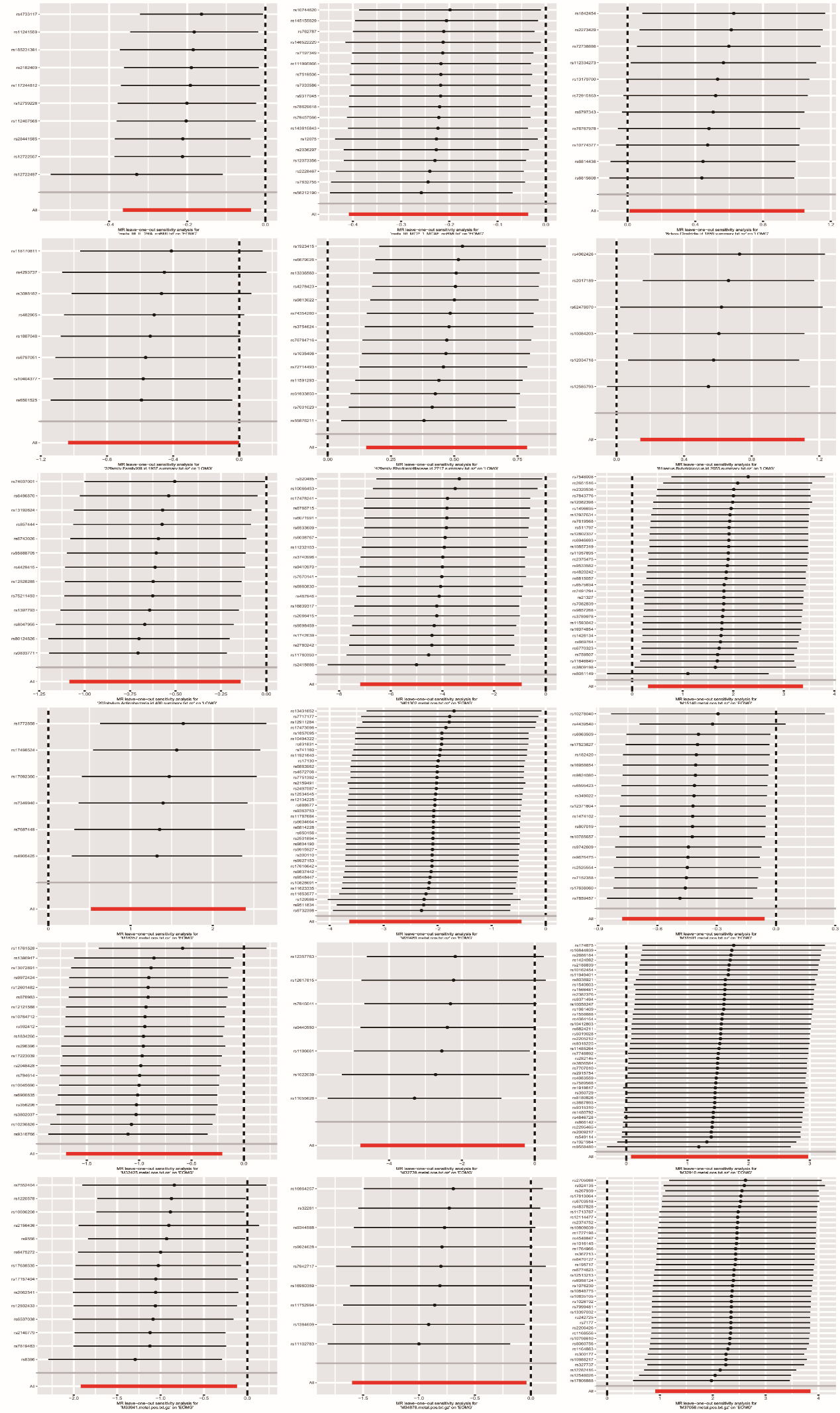


Supplementary Figure 3 Summary figures of LOO analysis results of significantly correlated exposure factors and LOMG.
